# Clinic Network Collaboration and Patient Tracing to Maximize Retention in HIV Care

**DOI:** 10.1371/journal.pone.0127726

**Published:** 2015-05-26

**Authors:** James H. McMahon, Richard Moore, Beng Eu, Ban-Kiem Tee, Marcus Chen, Carol El-Hayek, Alan Street, Ian Woolley, Andrew Buggie, Danielle Collins, Nicholas Medland, Jennifer Hoy, for the Victorian Initiative for Patient Engagement and Retention (VIPER) study group

**Affiliations:** 1 Department of Infectious Diseases, The Alfred Hospital and Monash University, Melbourne, Australia; 2 Infectious Diseases Unit, Monash Health, Melbourne, Australia; 3 Burnet Institute, Melbourne, Australia; 4 Northside Clinic, Melbourne, Australia; 5 Prahran Market Clinic, Melbourne, Australia; 6 Centre Clinic, Melbourne, Australia; 7 Melbourne Sexual Health Centre, Alfred Health, Melbourne, Australia; 8 Central Clinical School, Monash University, Melbourne, Australia; 9 Royal Melbourne Hospital, Melbourne, Australia; University of Athens, Medical School, GREECE

## Abstract

**Background:**

Understanding retention and loss to follow up in HIV care, in particular the number of people with unknown outcomes, is critical to maximise the benefits of antiretroviral therapy. Individual-level data are not available for these outcomes in Australia, which has an HIV epidemic predominantly focused amongst men who have sex with men.

**Methods and Findings:**

A network of the 6 main HIV clinical care sites was established in the state of Victoria, Australia. Individuals who had accessed care at these sites between February 2011 and June 2013 as assessed by HIV viral load testing but not accessed care between June 2013 and February 2014 were considered individuals with potentially unknown outcomes. For this group an intervention combining cross-referencing of clinical data between sites and phone tracing individuals with unknown outcomes was performed. 4966 people were in care in the network and before the intervention estimates of retention ranged from 85.9%–95.8% and the proportion with unknown outcomes ranged from 1.3-5.5%. After the intervention retention increased to 91.4–98.8% and unknown outcomes decreased to 0.1–2.4% (p<.01 for all sites for both outcomes). Most common reasons for disengagement from care were being too busy to attend or feeling well. For those with unknown outcomes prior to the intervention documented active psychiatric illness at last visit was associated with not re-entering care (p = 0.04)

**Conclusions:**

The network demonstrated low numbers of people with unknown outcomes and high levels of retention in care. Increased levels of retention in care and reductions in unknown outcomes identified after the intervention largely reflected confirmation of clinic transfers while a smaller number were successfully re-engaged in care. Factors associated with disengagement from care were identified. Systems to monitor patient retention, care transfer and minimize disengagement will maximise individual and population-level outcomes for populations with HIV.

## Introduction

Since the advent of combination antiretroviral therapy (ART) the morbidity and mortality of people living with HIV (PLHIV) has significantly improved [1–3]. In addition to the individual benefits of ART for PLHIV, there are population level benefits from ART through the reduction of transmission of HIV from individuals with suppressed HIV viral loads, namely treatment as prevention [4]. The recognition of both individual and population level benefits of ART has led to increasing interest in the cascade of care for HIV infection. Understanding the factors that contribute to retention of patients in HIV care and subsequent virological suppression in those receiving ART is therefore critical to maximising individual and population level benefits of ART [5, 6].

Latest estimates, as of 2013, are that 6300 people are living with HIV in Victoria, [7] and that median CD4 T-cell counts at diagnosis of HIV in Victoria are 454 cells/μL in men and 318 cells/μL in women, [7] representing on average an estimated 4 years between HIV infection and diagnosis [8]. Furthermore, in 2013 approximately 20% of PLHIV received their initial diagnosis with advanced HIV infection (measured by a CD4 T-cell counts below 200 cells/μL) while other individuals who are already aware of their diagnosis and have intersected with HIV services in the past are also represented with low CD4 T-cell counts [7].

Previous HIV care cascades, including the only Australian estimates, have often reported at an ecological level by combining different datasets and have not determined retention in care at the individual level [5–7, 9]. Individuals who have previously entered HIV care and are possibly lost to follow up, are also referred to as having ‘unknown outcomes’. Establishing whether people with ‘unknown outcomes’ have transferred care to another site, or are truly disengaged from care will allow for more accurate estimates of the true number retained in HIV care with access to ART. Accurate estimates of retention in HIV care will allow care providers and researchers to target steps in the HIV care cascade that harness the benefits of ART for PLHIV and individuals at risk of HIV infection. Therefore, this project sought to determine whether people who have been previously engaged in HIV care in Melbourne and now have ‘unknown outcomes’ have died, transferred their care to another site or become disengaged from care. Furthermore, this work sought to identify individuals with unknown outcomes who are subsequently able to re-engage in HIV care and reasons why this occurred.

## Methods

A clinical network of HIV care providers established for this study comprises the three high HIV caseload general practitioner (GP) clinics (Prahran Market Clinic, Northside Clinic, Centre Clinic), the Melbourne Sexual Health Centre (MSHC) and the two largest hospitals for the care of PLHIV (The Alfred, Monash Medical Centre). These sites are the major stakeholders in HIV clinical care in Victoria. All sites have electronic data systems that allow for the aggregation of data for people with the diagnosis of HIV via dedicated databases (Hospitals and MSHC) or via practice management software (GP sites).

The following steps were taken at all sites to identify PLHIV with unknown treatment outcomes. PLHIV at each site who have received HIV care from 1 March 2011 up until 31 May 2013 were identified based on one or more attendances where a HIV viral load test was performed. From this group of individuals it was established who did not have a viral load performed for the 9 months subsequent to 31 May 2013, therefore up until 28 February 2014. The 9 month period was selected as across all sites the longest interval routinely recommended for clinical review and assessment of HIV viral load is once every 6 months [10].

The medical records were reviewed at each site to establish study classifications for individuals without viral load in the 9 month period after 31 May 2013 (Table 1). Individuals were classified into different categories of retained in care if there was evidence of ongoing HIV care. Confirmed transfers of care required evidence of care occurring at another HIV site compared to unconfirmed transfers where the patient intended to transfer but there was no evidence that care was occurring elsewhere. Remaining people where there was no information of where care was occurring or whether they were alive were considered to have unknown outcomes.

**Table 1:** Study classifications after individual record review for people who had entered HIV care but with no viral load testing in the 9 months after 31 May 2013.

Classification	Description
Retained in care	Evidence of a viral load performed at an outside laboratory in the 9 month period
Retained with irregular viral load	Evidence of ongoing contact, including prescribing and dispensing of ART, within the 9 month period but without a viral load test performed
Retained at an external site	Despite HIV viral load occurring at the site never attended the site for HIV care and evidence that receiving HIV care at another site. Definition most applicable to hospitals where PLHIV could be admitted and have viral load tests performed while receiving non-HIV care
Shared care	Evidence that attends more than one 1 site regularly for HIV care. Mainly applicable to people attending a primary care and a hospital site
Died	Evidence person had died
Confirmed transfer	Evidence of receiving HIV care from another HIV service provider (e.g. transfer of medical records request, medical correspondence, results) including the name of the site
Unconfirmed transfer	Evidence that planned for transfer or ongoing care elsewhere but no formal documentation to confirm transfer occurred
Unknown	No information of where care was occurring or whether person was alive

For individuals classified as ‘Unknown’ or ‘Unconfirmed transfer’ partially de-identified information (4 letter name code [first 2 letters of last and first name] and date of birth) was shared with other sites in the clinical network to see if further outcomes could be determined, principally whether they had transferred care. These data were also cross-referenced with the HIV notification registry maintained at the Burnet Institute, which collects details on deaths for PLHIV in Victoria

For remaining individuals still considered ‘Unknown’ or ‘Unconfirmed transfer’ then the site where the patient last attended for HIV care attempted to contact these individuals using a standardised phone script. Patients who were contacted and not in HIV care were invited to re-engage in HIV care at the original site or another HIV care site and were considered to be ‘Re-engaged to care’ if they indeed attended for HIV care. Individuals who spontaneously returned to care in the process of cross-referencing data between sites and performing the phone tracing in the latter half of 2014 were classified as ‘Returned to care’. The definition of the intervention for this study is the combination of: i) cross-referencing data between sites, and ii) phone tracing.

Site-level estimates of retention in care, transfer of care and unknown outcomes are expressed as proportions of the total number of people who have accessed HIV care at the site over the period from 1 March 2011 to 31 May 2013. Individuals considered not to have entered HIV care (i.e. classified as “Retained at an external site’) are excluded from the denominator for these estimates.

Individuals who could be contacted for phone tracing and had experienced interruption in care, were asked about reasons why they may have interrupted using a standardized questionnaire including: feeling well, being too busy to attend, financial barriers, issues with transport to clinic, and any additional factors which may have contributed to care interruption.

In addition, data from medical records for patients with unknown outcomes who remained disengaged from care were compared to those with unknown outcomes who transferred care or returned to care. These data included: demographic factors, access to Medicare (the publicly funded health system providing care and ART at no or minimal cost), receiving ART at last visit, viral load at last visit, non-English speaking background (born outside Australia and first language not English) and active psychiatric illness at last visit (on medication for psychiatric illness or documented as symptomatic). McNemar’s test was used to compare categorical outcomes in paired groups. In unpaired groups Chi-squared or Fisher’s exact test were used to compare categorical outcomes, Student’s t-test to compare continuous outcomes and Wilcoxon rank sum test to compare continuous outcomes with non-normal distribution. Statistical tests were conducted using Stata software (Version 12, College Station, TX, U.S.A.). The ethical review boards at the Alfred and Monash Medical Centre approved the study for all sites.

## Results

**Fig 1:**
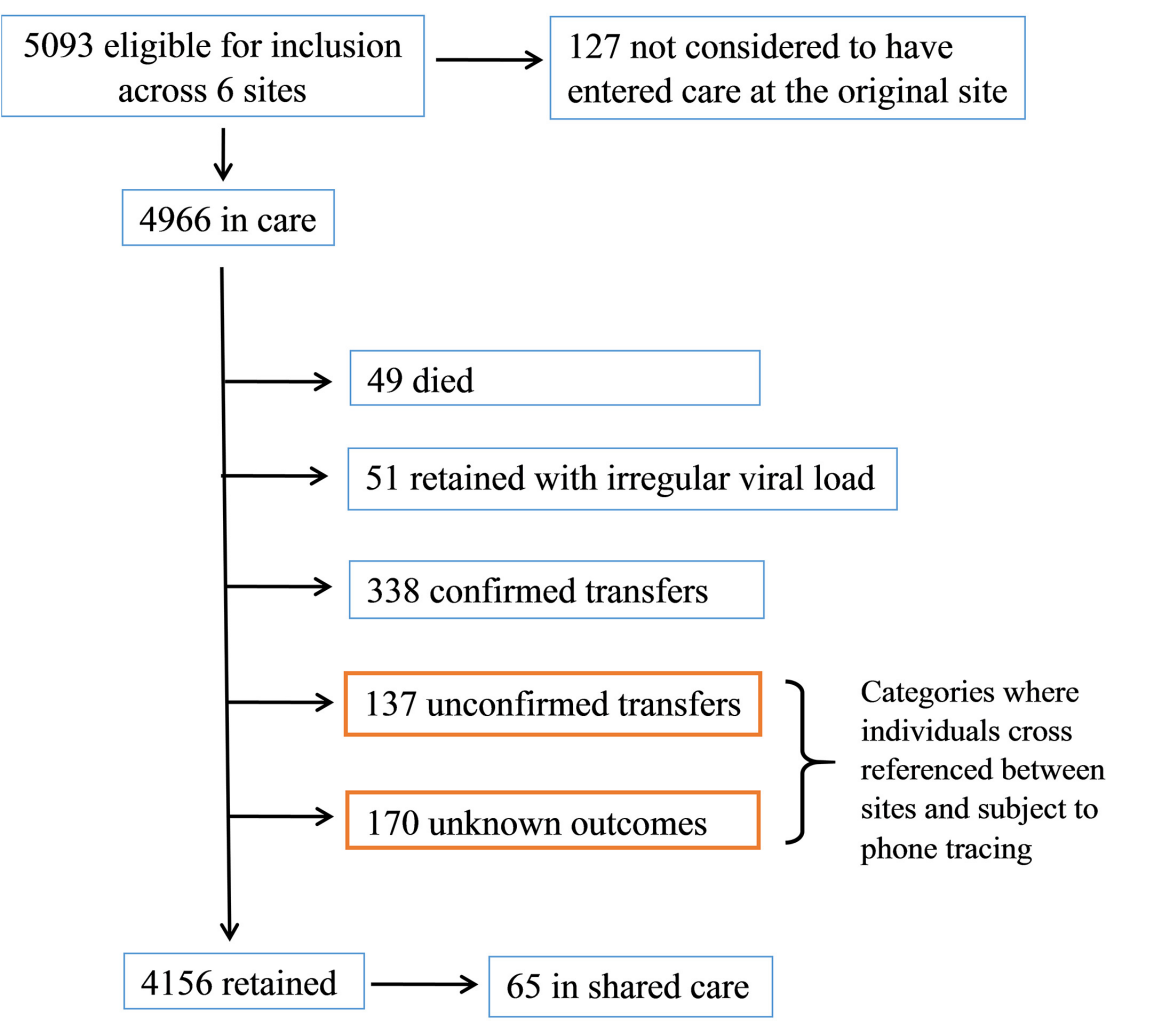
Consort diagram of total numbers of patients entering analysis.

Across 6 sites 5093 individuals had HIV viral loads performed during the period 1 March 2011 to 31 May 2013. Of these, 127 individuals, 119 at hospital sites and 8 at the sexual health centre, were classified as 'Retained in care at external site, and were excluded. This left 4966 individuals considered in care at their respective sites. (Fig 1).

**Fig 2:**
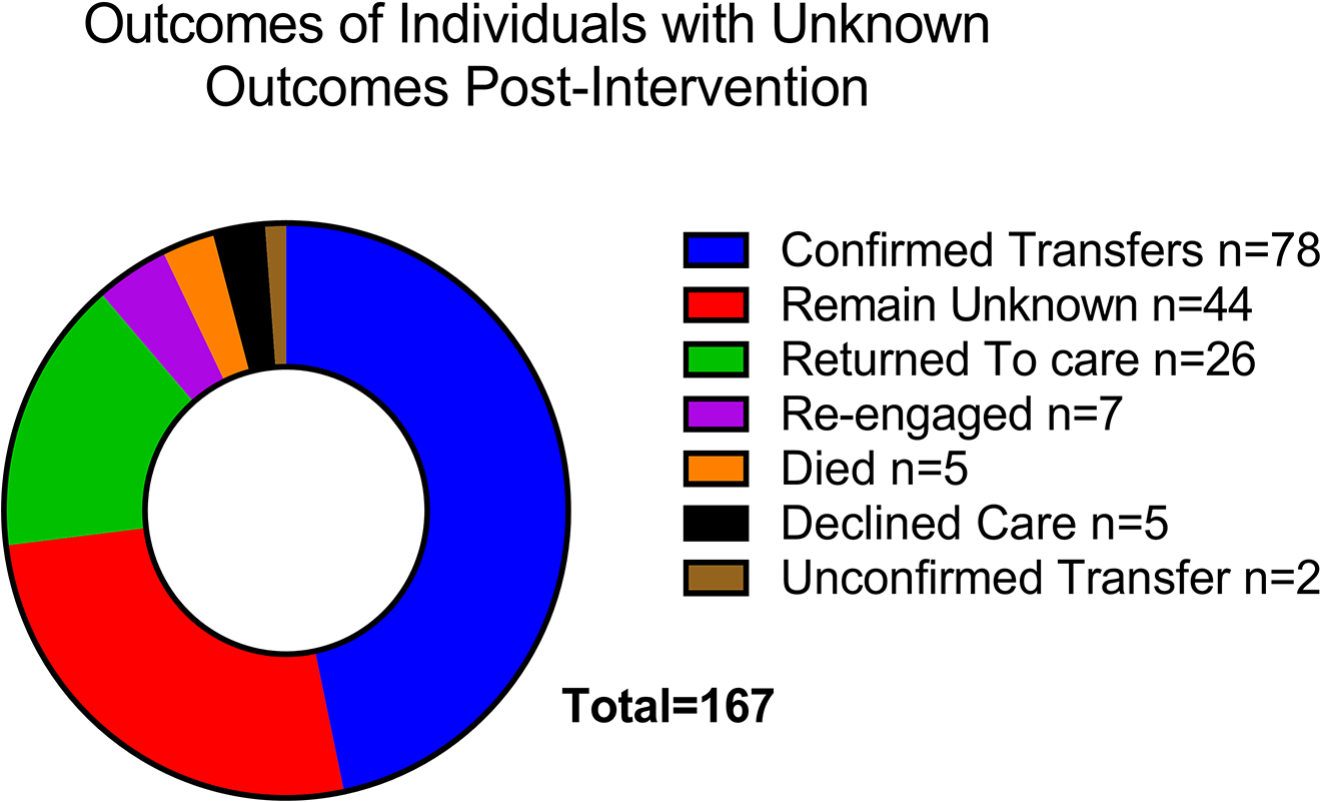
Status of individuals post-intervention who were originally considered to have unknown outcomes.

Estimates of unknown outcomes, unconfirmed transfer, confirmed transfer and retention are summarised in Table 2. The proportion with unknown outcomes before the intervention ranged from 1.3–5.5% across the sites and dropped to 0.1–2.4% after the intervention; (p<.01 at all sites). Final outcomes after the intervention for the group with unknown outcomes is detailed in Fig 2. Unknown outcomes were more similar at specialist GP clinics (1.4–2.8% pre- and 0.1–1.1% post-intervention) while the combination of hospitals and sexual health centre had similar proportions with unknown outcomes (4.9–5.5% pre- and 1.2–2.4% post-intervention). (p<.01 for comparison of GP to non-GP sites pre and post intervention). The intervention also resulted in reductions in unconfirmed transfer across all sites, reaching statistical significance (p<.05) in all but one GP site. Confirmed transfers pre-intervention ranged from 1.6–10.9% and post-intervention from 3.6–14.4%. (p<.01 at all sites). Greater increases were seen at hospital and sexual health centre sites consistent with the greater reduction in unknown outcomes at these sites. Estimates of retention in care that categorised confirmed transfers as still being retained in care ranged from 85.9–95.8% and rose to 91.4–98.8% post the intervention (p<.01 at all sites). Retention in care estimates that did not consider confirmed transfers as retained had non-significant increases apart from the sexual health centre site, 80.1–92.0% to 81.2–92.5%.

**Table 2:** Key outcomes by site.

			Outcome									
		Individuals in care^a^	Unknown^b^ n (%)		Unconfirmed transfer^c^ n (%)		Confirmed transfer^d^ n (%)		Retention^e^ %		Retention inc. confirmed transfer^f^ %	
Pre- or Post-intervention			Pre	Post	Pre	Post	Pre	Post	Pre	Post	Pre	Post
**Site**	**SPC 1**	805	11 (1.4)	1 (0.1)^#^	23 (2.9)	6 (0.7)^#^	30 (3.7)	51 (6.3)^#^	92.0	92.5	95.8	98.8^#^
	**SPC 2**	1102	14 (1.3)	4 (0.5)^#^	40 (3.6)	32 (2.9)^#^	25 (2.3)	40 (3.6)^#^	89.9	90.1	92.2	93.7^#^
	**SPC 3**	464	13 (2.8)	5 (1.1)^#^	5 (1.1)	1 (0.2)	39 (8.4)	49 (10.6)^#^	84.7	84.7	93.1	95.3^#^
	**TMC 1**	1188	61 (5.1)	13 (1.2)^#^	23 (1.9)	11 (0.9)^#^	114 (9.6)	161 (13.6)^#^	80.6	81.2	90.2	94.8^#^
	**TMC 2**	255	14 (5.5)	6 (2.4)^#^	12 (4.7)	5 (2.0)*	4 (1.6)	16 (6.3)^#^	84.3	85.1	85.9	91.4^#^
	**SHC**	1152	57 (4.9)	18 (1.7)^#^	34 (3.0)	16 (1.4)^#^	126 (10.9)	166 (14.4)^#^	80.1	81.3^#^	91.1	95.7^#^

**NOTES:** SPC, specialist primary care; TMC, tertiary medical centre; SHC, sexual health centre

* p<.05 for comparison to pre-intervention figure (McNemar’s test)

^#^ p<.01 for comparison to pre-intervention figure (McNemar’s test)

^a^ individuals with at least one HIV viral load from 1/3/2011 to 31/5/2013 at the site excluding individuals who had not received HIV care at the site and were known to be in HIV care at an external site

^b^ Individuals with unknown outcomes

^c^ Individuals thought to have transferred care but no evidence in medical records to confirm that transfer occurred

^d^ Evidence in medical records that care was transferred

^e^ Individuals in care at the site or sharing with another site as a proportion of all individuals in care

^f^ Defined as for retention but considers confirmed transfers also retained in care

In addition 32 individuals either returned to care or were re-engaged in HIV care after an interruption with 29 individuals providing information on why care was interrupted (Table 3). The most common reasons reported were feeling well (n = 12) and being too busy (n = 7). A range of other reasons were reported including psychosocial stressors, barriers to attending clinic and difficulty with accepting the diagnosis of HIV. Notably no individuals reported physical symptoms or problems from ART as being reasons for interruption.

**Table 3:** Reported reasons for interruption of HIV care (n = 29).

Reason Category	Specific Reason^a^	Number of times reported
Well and Busy	Felt well^b^	12
	Longterm non-progressor “so won’t get sick"	1
	Too busy^b^	7
Structural Barriers	Difficulty attending clinic^b^ (e.g. transport, parking, long wait time)	5
	Difficulty arranging review	1
	Financial^b^	2
Psychosocial	Psychosocial stressors (unspecified)	5
	Difficulty accepting HIV diagnosis	2
	Wanting to ignore HIV	2
	Apathy and lowered mood	1
Other	Wanted a break from care	2
	Overseas for extended period	2
	Negative interaction with site	1
	Ran out of ART	1
	Incarcerated	1
	Don’t believe in conventional treatments	1
	Needle phobia	1

NOTES:

^**a**^ Despite being specific reasons listed on the questionnaire no individuals reported physical symptoms or problems from ART contributing to interruption

^b^ Specific reasons listed on the questionnaire

When examining baseline factors of 53 people who remained unknown compared to the 111 who returned to care after the intervention being treated for a psychiatric condition or presence of symptoms of a psychiatric illness was present in 55.6% of people who remained with unknown outcomes compared to 20.7% without this classification (p = 0.04). (Table 4) Those of a non-English speaking background more often remained with unknown outcomes versus those with an English speaking background, 32.5% versus 17.1% (p = 0.26), while access to publicly funded Medicare was more common in those returning to care, 97.3% versus 92.5% (p = 0.21). People remaining with unknown outcomes post the intervention were also less likely to be receiving ART and have higher viral loads at their last visit with a trend to statistical significance, 49.1% versus 60.4% (p = 0.17) and median viral load 600 copies/mL versus 99 copies/mL (p = 0.15) respectively.

**Table 4:** Characteristics of individuals with unknown outcomes compared for those whose outcomes remain unknown, died or declined care post-intervention^a^ to those who re-entered or transferred care.

Characteristic	People with unknown outcomes pre-intervention^a^ (n = 164)	Re-entered or transferred care (n = 111)	Remained unknown, died or declined (n = 53)	P value
Age (± SD)	39.9 ± 9.8	40.9 ± 10.2	38.3 ± 8.8	0.3
Gender (% male)	91.5%	90.1%	92.5%	0.8
Transmission risk category (% MSM)^b^	67.0%	71.8%	58.5%	0.11
Non-English speaking background^c^	24.2%	17.1%	32.5%	0.26
Medicare card holders^d^	95.7%	97.3%	92.5%	0.21
Receiving ART at last visit	56.7%	60.4%	49.1%	0.17
Active psychiatric condition^e^	25.6%	20.7%	55.6%	0.04
Viral load copies/mL (Median, IQR)	127 (UD—21212)	99 (UD—18300)	600 (UD—46100)	0.15

**NOTES:** MSM, men who have sex with men; ART, antiretroviral therapy; UD, undetectable

Characteristics compared by χ2 test or Fisher’s exact test if cell frequencies ≤5 apart from age (students’ t-test) and viral load (Wilcoxon rank sum test)

^a^ Intervention of cross referencing data between sites and phone tracing for those still with unknown outcomes

^b^ MSM as compared to non-MSM categories (IDU, combined IDU/MSM, Heterosexual, Unknown)

^c^ Born outside of Australia and first language is not English

^d^ Holder of Medicare card that allows access to publicly funded healthcare

^e^ Receiving medication for a psychiatric condition (e.g. depression, anxiety, schizophrenia, bipolar affective disorder) or documented symptomatic psychiatric condition at last visit

## Discussion

This study describes low proportions of people with unknown outcomes and high levels of retention in 2014 for people who in HIV care at the 6 largest sites in Victoria, Australia. Estimates of retention from 85.9%-95.8% increased to 91.4–98.8% after an intervention combining cross-referencing data between sites and phone tracing. In addition, while this project did not seek to determine the proportion receiving ART or virologically suppressed these retention data sit well with national and local data reporting 87–93% of PLHIV in care receiving ART and 89–94% of those virologically suppressed [7, 11]. Results of this study also compare favourably to other high income country settings where HIV epidemics are characterized by a high proportion of men who have sex with men (MSM) making up the population living with HIV as in Australia [9, 12–15]. For example, countries with publicly funded health systems and high quality of life indices [16] such as Denmark, Sweden, France and Belgium report retention of individuals already linked to care of 90–92% [12, 13, 15]. In North America, retention of those already in care is 85–90% in Canada where care is publicly available [14] whereas in the United States 66% of MSM who are linked to care are retained [9].

Findings in our study likely reflect that people with HIV are able to access and maintain themselves in HIV care in the publicly funded Australian health system. Importantly people in this study do not pay to see the doctor or to have blood tests at the hospital sites or sexual health centre, and people at specialist GPs who hold concession cards they also receive these services for free. Costs for antiretroviral therapy vary but most commonly those attending specialist GPs and the sexual health centre have no costs [17] but those at hospital pay a median annual cost of $216.60 if a non-concession card holder or $59.00 if a concession card holder [18].

The intervention to cross-reference data between sites and trace individuals with unknown outcomes led to reductions in unknown outcomes and increases in retention. Most of this improvement was due to the reclassification of individuals as confirmed transfers as they had been determined to attend care elsewhere after cross-referencing data. The approach of sharing individual level data across services and carefully cross matching allows for more accurate ascertainment of patient outcomes. This method has distinct advantages in comparison to estimates of retention that use ecological data or link datasets from different groups without the ability to link individual-level data [5–7, 9]. Individual case investigation of those potentially disengaged from care is an opportunity to re-introduce PLHIV to the benefits of ART and also ensures available data does not over-represent loss-to-follow-up and under-estimate retention in care or community coverage of ART [19, 20], Notably current reported estimates of retention in Australia are assumed from data about the number of CD4 tests ordered in Australia combined with CD4 test ordering patterns in an observational cohort of people with HIV [7]. The phone tracing component of the intervention led to a modest number of people re-engaged in care but this activity was impacted by having inaccurate or outdated contact information. Notably we did not attempt to contact listed next of kin due to concerns about divulging personal information. The inability to contact lost patients emphasises the importance of updating contact information at all sites and considering alternate communication methods such as email to facilitate future tracing efforts.

An additional advantage of this work is that it highlights different models of care at different sites and their impact on estimates of retention and disengagement. Many individuals had HIV viral load testing at hospital sites but received no HIV care there. This includes examples where these people attended hospital for care of other medical conditions and during that presentation HIV viral load testing was performed potentially for the benefit of another care provider or for unclear reasons. These individuals were classified as ‘retained in care at an external site’ after medical record review established evidence of HIV care elsewhere. This finding highlights the importance of not making assumptions about retention or disengagement from HIV care based on laboratory testing alone but incorporating clinical review of cases where people are thought to have unknown outcomes. This method of classification allowed these individuals to be removed from the denominator of calculations of retention and unknown outcomes so more accurate estimates could be obtained. An additional insight into different models of care delivery comes from lower levels of unknown outcomes at primary care sites prior to implementing the intervention. This may reflect more stable populations in primary care or record keeping systems that are better at determining if patients with unknown outcomes are in fact transferred.

Reasons for disengagement varied but did not focus on poor health or adverse effects of medication but most commonly on being ‘well and busy’ which has been reported outside Australia [21]. Other important areas are structural barriers to care such as transport and finances with the other major category being different psychosocial stressors or issues around acceptance of diagnosis. These responses highlight the importance of maintaining an awareness of the need for ongoing HIV care particularly in people that are clinically well and allowing flexibility for people to attend HIV care with busy schedules or have difficulty attending at certain times due to issues obtaining transport. Further, individuals experiencing psychological stress are also at risk, factors that have been reported elsewhere [22, 23]. Analysis of baseline factors predicting re-engagement to care also identified active psychiatric illness as associated with disengagement. Other factors such as non-English speaking background and lack of access to publicly funded Medicare services also were more common in those who remained disengaged. This emphasizes how marginalised groups are at risk for poor clinical outcomes and points to targets for intervention to improve retention in HIV care.

Potential limitations of this analysis include the definition for whether individuals were eligible for consideration as having unknown outcomes. We used a definition of not having an HIV viral load in a 9-month period after initially entering care. Possibly this definition would be too lax for some individuals with unknown outcomes who did have a single viral load early in the 9-month period then subsequently were lost to care. Conversely a shorter time-frame, for example 6 months may have considered stable individuals who only attend for clinical review 6 monthly as having unknown outcomes. Notably current treatment guidelines recommend 6-monthly viral load monitoring for stable adherent virologically suppressed patients [10]. It is also important to consider that this analysis does not make distinction between people initiating HIV care versus those already in ART care. Outcomes for these groups are potentially different, for example, worse outcomes for those initiating care as compared to those who have established care over many years. In addition the sites included in this study are the largest sites for HIV care in the state caring for over 75% of PLHIV in the state. Therefore there is the potential for a selection bias as smaller sites with less capacity or experience in HIV care may have different outcomes than the data reported here.

In conclusion, these six sites accounting for over 75% PLHIV in Victoria demonstrated low numbers of people with unknown outcomes and high levels of retention in care. Furthermore, the low-cost methods used to determine these outcomes, re-engage patients in care and identify factors associated with disengagement are feasible and replicable over time. Increased levels of retention in care and reductions in unknown outcomes identified after the intervention largely reflected confirmation of clinic transfers while a smaller number were successfully re-engaged in care. Systems to monitor patient retention, care transfer and minimize disengagement will enable an accurate understanding of the number PLHIV with unknown outcomes and maximises individual and population-level outcomes for PLHIV.
